# Molecular aspects of Angelman syndrome: Defining the new path forward

**DOI:** 10.17305/bb.2025.11724

**Published:** 2025-03-17

**Authors:** Jacqueline Fátima Martins de Almeida, Ilaria Tonazzini, Simona Daniele

**Affiliations:** 1 Department of Pharmacy, University of Pisa, Pisa, Italy; 2 NEST, Nanoscienze Institute-CNR and Scuola Normale Superiore, Pisa, Italy

**Keywords:** Angelman syndrome, AS, epigenetic repression, genetic imprinting disorders, neuronal plasticity, ubiquitin-protein ligase E3A, UBE3A, silencing mechanism

## Abstract

As a rare neuro-genetic disease, Angelman syndrome (AS) affects about 15 to 500 thousand people worldwide. The AS is an imprinting genomic disease characterized by the loss of function of the maternal *UBE3A* gene, located in the 15q11-q13. This gene encodes a ∼100 kDa protein, the Ubiquitin-protein ligase E3A (UBE3A), that participates in the ubiquitination process, one of the post-translational protein modifications. In the brain, under normal conditions, the paternal allele of the *UBE3A* gene is silenced, with only the maternal allele being active. However, in individuals with AS, the maternal loss of function of this gene leads to the complete absence of UBE3A expression, resulting in multiple pathological features. Clinically, children diagnosed with AS exhibit a characteristic behavioral phenotype, including a happy demeanor, frequent and unmotivated laughter, movement, speech impairment, severe intellectual disability, and sleep problems. Since its discovery in 1965, significant progress has been made in understanding the genetic and pathophysiological aspects of AS. However, despite these advances, the molecular mechanisms underlying the disease remain incompletely understood, and no effective treatment currently exists. Current therapies focus solely on symptom management, and no approach has yet succeeded in reactivating the silenced paternal *UBE3A* allele. Therefore, this review highlights the epigenetic aspects involved in the AS in order to provide a better understanding and clarification of the mechanisms, hopefully paving the way for future research to improve the treatment of affected individuals.

## Introduction

Neurological conditions are the leading cause of illness and disability worldwide [1]. In 2021, more than three billion people suffered from neurological disorders [2]. Among these, neurogenetic disorders—including neurodevelopmental disorders (NDDs)—represent one of the most significant and challenging groups.

NDDs encompass a diverse range of conditions that typically manifest early in life and are primarily associated with neurodevelopmental impairments. In 1965, Angelman syndrome (AS) was added to this group. AS is caused by the loss of function of the maternally inherited *UBE3A* gene, located in the 15q11-q13 chromosomal region [3, 4]. The *UBE3A* gene encodes *ubiquitin-protein ligase E3A*, a ∼100 kDa protein involved in ubiquitination, a key post-translational modification [3].

The loss of a functional *UBE3A* gene directly and indirectly contributes to several pathological features. Although children with AS typically have a normal prenatal and birth history, as well as normal laboratory parameters, developmental delays become noticeable only around six months of age [5]. Many clinical features of AS overlap with characteristics of other NDDs, such as movement and balance disorders, speech impairments, and behavioral abnormalities. As a result, diagnosis is often delayed until approximately 12–20 months of age [6].

Significant advances in understanding the genetic aspects of this disease have been made since its discovery in 1965. Notably, in 1984, researchers identified AS as a striking example of genomic imprinting—an epigenetic phenomenon in which a gene is expressed from only one parental allele. Under normal conditions, the paternal allele of the *UBE3A* gene is silenced, leaving only the maternal allele active. However, in individuals with AS, a loss of function in the maternal allele prevents *UBE3A* expression.

**Table 1:** Overview of clinical features and genetic cause of AS patients, from studies with large cohort published in the last 5 years

**Characteristics|Authors**	**Du et al., 2024** **(PMID: 36011358)**	**Carriero et al., 2024** **(PMID: 38930051)**	**Bindels-de Heus et al., 2019** **(PMID: 31729827)**	**Den Besten et al., 2020** **(PMID: 33108066)**	**Manoubi et al., 2024** **(PMID: 38322471)**
Total of patients	695	62	100	95	50
Mean or range of age (months)	6.34 ± 2.94	8.0 ± 17.7	5.7 ± 4.8	31.6 ± 12.6	12–84 months
Country of the study	China	Italy	Netherlands	Netherlands	Tunisia
Age at diagnosis (months)	31.7 ± 24.1 4	24 ± 11.4	30 ± 27.6	NR	NR
*Symptoms*					
Epilepsy	554 (79.7%)	51 (82.2%)	82 (82%)	84 (89.4%)	44 (88%)
Sleep problem	613 (88.2%)	43 (69.4%)	91 (91%)	81 (88%)	45 (90%)
Feeding problems	564 (81.2)	40 (64.5%)	45 (45%)	45/91 (49%)	47 (94%)
Speech impairment	695 (100%)	49 (79%)	NR	95 (100%)	40 (80%)
Strabismus	375 (54%)	42 (67.8%)	40 (40%)	30 (32%)	NR
Behavioral features	647 (93.1%)	57 (92%)	NR	NR	48 (96%)
*Genetic cause*					
Deletions	577 (83%)	36 (58%)	62 (62%)	56 (58.9%)	NR
Non-deletions mutations	118 (17%)	26 (42%)	38 (38%)	39 (41.1%)	NR 7 (14%)

NR: Nonreported; AS: Angelman syndrome.

Genetic imprinting is just one example of many epigenetic phenomena, in this case modulated by DNA methylation. In the *UBE3A* gene region, located on chromosome 15q11-q13, an imprinting center (IC) situated 35 kb upstream of the *SNURF-SNRPN* promoter regulates the imprinting area through DNA methylation. This process may be coordinated by the long noncoding antisense RNA *SNHG14* [7]. The imprinted domain on human chromosome 15 consists of two oppositely imprinted gene clusters, both under the coordinated control of the IC at the 5’ end of the *SNURF-SNRPN* gene. In this way, the maternal-only expression of *UBE3A* may be regulated indirectly through a paternally expressed antisense transcript. Specifically, a processed antisense transcript of *UBE3A* originates at the IC. The *SNURF-SNRPN sense/UBE3A* antisense transcription unit contains at least 148 exons, including the previously identified IPW exons (e.g., HBII-13, HBII-85, and HBII-52 snoRNAs), as well as four additional snoRNAs: HBII-436, HBII-437, HBII-438A, and HBII-438B [3, 7, 8].

Despite significant progress in understanding the molecular complexity of this disease, it remains a puzzle, and, unfortunately, no effective treatment currently exists. Current therapies focus solely on managing symptoms, and there is still no known method to reverse the imprinting of the paternally silenced gene. This review aims to summarize the molecular aspects of AS, emphasizing the lack of sufficient epigenetic research in this area. By highlighting these gaps, we hope to pave the way for future studies that could lead to improved treatments for affected individuals.

### Clinical aspects

Neurogenetic disorders encompass a wide range of diseases that arise during nervous system development. The overlapping clinical features among NDDs contribute to a broad differential diagnosis, encompassing at least 13 neurological diseases, which makes early and precise diagnosis challenging [9, 10].

In 1965, a new neurological disorder was identified when an English physician, Harry Angelman, observed three unrelated children with similar characteristics, including flat heads, jerky movements, protruding tongues, and frequent bouts of laughter. During a vacation in Italy, Angelman encountered an oil painting titled A Boy with a Puppet, which reminded him of these children. Inspired by this, he published the first description of the condition, initially referring to the affected patients as “puppet children.” The disorder was later named (AS) in his honor [11].

AS is a rare neurogenetic disorder affecting approximately 15,000 to 500,000 people worldwide (Angelman Syndrome Foundation). Reports in the literature suggest a low incidence rate, ranging from 1 in 10,000 to 1 in 24,000 [12, 13].

Despite overlapping clinical features with other neurological disorders, the most characteristic findings in individuals with AS include severe developmental delay by 6–12 months of age, delayed achievement of developmental milestones without regression, absence of speech, epilepsy, sleep disturbances, gastrointestinal issues, a fascination with water, and a consistent behavioral phenotype. This phenotype is marked by a happy demeanor, easily provoked laughter, and hypermotoric behavior (Table 1) [4, 14–16].

Diagnosing AS is challenging due to overlapping clinical characteristics, with diagnosis typically occurring between 12 and 30 months (Table 1) [14–21]. Non-invasive prenatal tests for microdeletions, particularly for AS, have low sensitivity and positive predictive value, presenting critical limitations that hinder early diagnostic certainty [22].

### Genetic aspects

In 1987, in separate studies, Lawrence Kaplan and Ellen Magenis [23, 24] observed a deletion on the long arm of chromosome 15 in patients with AS, suggesting a potential genetic cause for the disease. This same deletion had already been identified in another genetic disorder, Prader-Willi syndrome (PWS).

**Figure 1.**
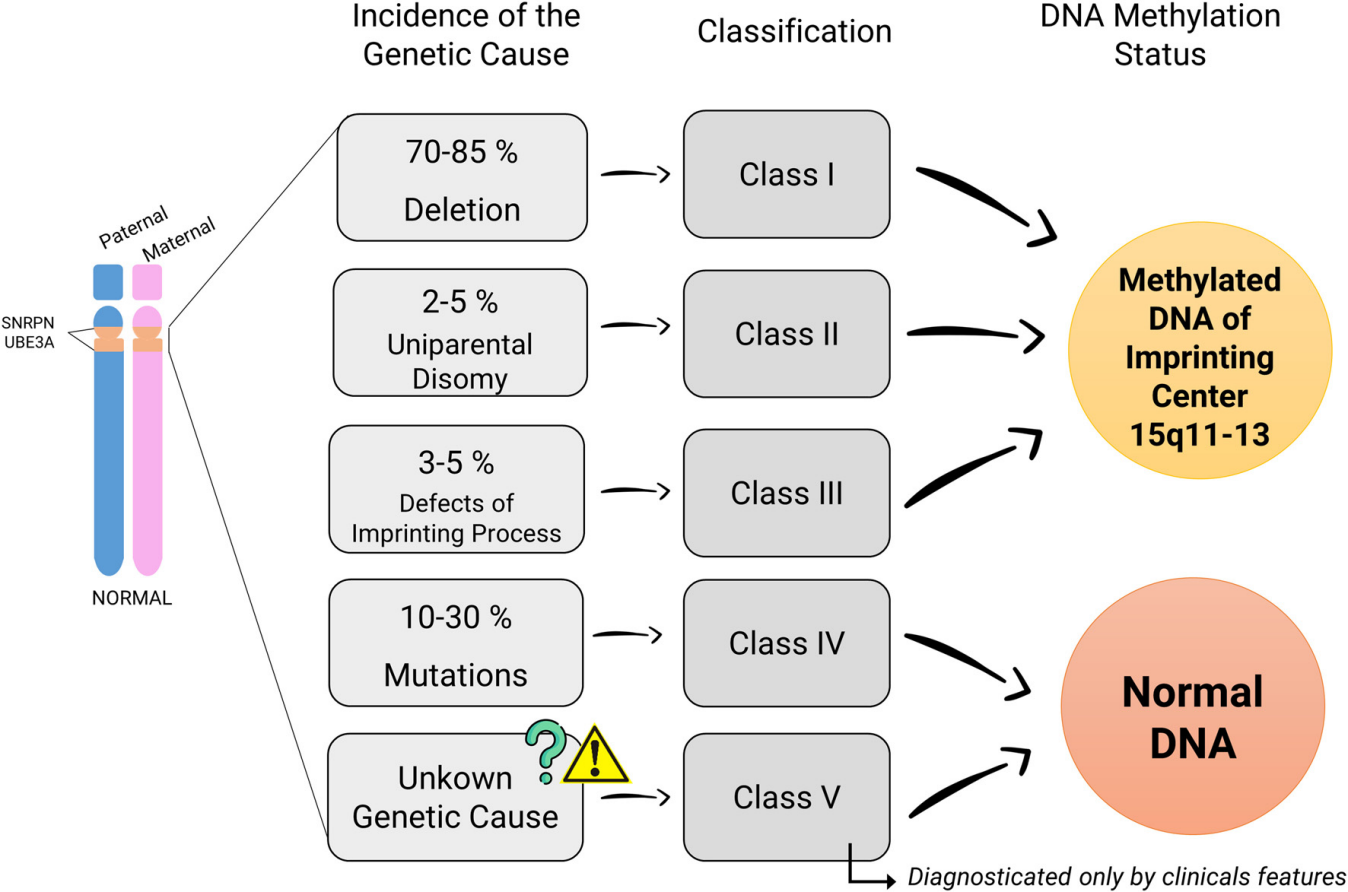
**Genetic cause distribution in Angelman syndrome with their clinical classification by the DNA methylation status:** Classes I–III typically show abnormal DNA methylation, while Class IV and V present normal methylation patterns.

By the late 1980s, studies on a small cohort of patients suggested a possible maternal origin of AS [25–28]. This was confirmed in 1992 by Smith and colleagues in a larger cohort of 25 individuals, all of whom exhibited a maternal inheritance pattern. Their findings established that PWS results from the loss of part of chromosome 15 from the paternal lineage, whereas AS arises from the loss of the same chromosomal region but from the maternal lineage [29]. Finally, in 1997, Kishino et al. [30] identified the gene responsible for AS: the *UBE3A* gene, which encodes the E6AP-E3 ubiquitin-protein ligase.

The 15q11-q13 region, which contains all the genes involved in both PWS and AS, is regulated by genomic imprinting and is known as the IC. Genomic imprinting is an epigenetic phenomenon in which a gene is expressed from only one allele, depending on its parental origin. These two syndromes serve as striking examples of imprinting disorders: the loss of the paternal chromosome leads to the clinical features of PWS, while the loss of the maternal chromosome results in AS.

The gene *UBE3A* is biallelically expressed in non-neuronal cells, whereas in neuronal cells, only the maternally inherited allele is expressed [31]. This imprinting pattern is regulated by the *UBE3A* antisense transcript, formerly known as *UBE3A-ATS* and now referred to as *SNHG14*, which silences the paternal allele specifically in neuronal cells. This mechanism will be discussed in more detail in the following section [31].

Initially, the deletion of 15q11.2 was thought to be the sole cause of AS. However, by 1990, studies revealed that not all patients exhibited this chromosomal deletion, suggesting the presence of additional molecular causes [32, 33].

It is now well known that AS has four molecular causes related to maternal loss of chromosome 15q11-q13. The most common is a de novo deletion of approximately 4 Mb in this region, occurring in 70%–85% of cases (patients classified as Class I) [30, 34]. The second most frequent cause involves intragenic mutations in the *UBE3A* gene (Class IV), accounting for 10%–30% of cases [35–38]. Less common causes include paternal uniparental disomy (UPD), present in 2%–5% of cases (Class II), and defects in the imprinting process, occurring in 3%–5% of cases (Class III) [4, 30, 39, 40]. Additionally, a fifth group of patients (Class V) does not fit into any of these categories. While they exhibit the main clinical features of AS, their genetic cause remains unidentified [40]. Unlike patients in Classes I–III, those in Classes IV and V show normal DNA methylation patterns, which can be confirmed through DNA methylation analysis of the 15q11-q13 IC [40, 41] (Figure 1).

The classification of patients based on their molecular status appears to influence the clinical course and progression of the disease. Given the complexity of the molecular mechanisms involved in AS, accurate classification is crucial for clinicians to better understand its clinical features and for researchers to develop effective treatments.

To achieve this, an AS diagnostic algorithm is used, beginning with DNA methylation analysis of the 15q11-q13 region. If the methylation pattern is normal, a mutation test is performed to classify patients into either Class IV (*UBE3A* mutation) or Class V (unknown cause). If DNA methylation is abnormal, further analysis is conducted using FISH or microarray techniques to detect microdeletions, which are characteristic of Class III patients (imprinting defect). If no microdeletion is found, DNA marker testing for UPD is performed to identify Class II patients (UPD) [16].

### Molecular epigenetics of AS

Epigenomic signatures include histone variants and modifications, alterations in nucleosome positioning, DNA methylation, and non-coding RNAs (ncRNAs) [42]. The first study to suggest a possible gender influence on offspring’s genetic inheritance was published in 1984 [43, 44]. In this work, Davor [43] and James McGrath, along with Surani et al. [44, 45], independently tested embryos containing either two sets of chromosomes inherited exclusively from the father or the mother. These embryos were transferred into pseudo-pregnant recipient females but failed to develop to term. This experiment demonstrated that although the chromosomes were genetically identical, they were not functionally equivalent without the presence of the opposite parental origin. Thus, normal embryonic development requires one set of chromosomes from each parent [46]. This phenomenon, known as genomic imprinting, refers to epigenetic inheritance in which gene regulation is influenced by parental origin. Offspring inherit an imprinted markercalled the gametic differentially methylated region (gDMR). This term was first used in 1991 when the first imprinted genes—*Igf2r*, *Igf2*, and *H19*—were discovered [47–50]. The differentially methylated region (DMR) inherited from a parent directs parental-specific allelic expression and is referred to as the IC. In genomic imprinting disorders, such as PWS and AS, DNA methylation plays a crucial role in maintaining the complexity of imprinting [8].

In 1992, it was discovered that the well-conserved region of the D15S63 locus in 15q11-q13 is methylated on the maternally inherited chromosome in PWS but remains unmethylated on the paternally inherited chromosome. In contrast, the opposite pattern occurs in AS [51, 52], making DNA methylation a valuable diagnostic marker for classifying AS patients [40]. Methylationat the IC of chromosome 15 suppresses gene expression, leading to gene silencing. Therefore, identifying the specific gene or gene cluster within the DMR is crucial for understanding the epigenetics of imprinting disorders [7].

In healthy individuals, the *UBE3A* gene is exclusively expressed from the maternal allele in the brain, while the paternal allele is silenced through genomic imprinting. This silencing is regulated by the bicistronic *SNURF-SNRPN* gene and orchestrated by the long noncoding antisense RNA *SNHG14* (formerly, *UBE3A-ATS*) [7, 53]. On the maternal chromosome 15q11-q13, the PWS-IC region is methylated. This epigenetic modification prevents transcription factors from binding to the promoter, thereby silencing the gene. In contrast, the paternal allele remains unmethylated, allowing the *SNURF-SNRPN* gene to transcribe lncRNA *SNHG14*, which contributes to *UBE3A* silencing [54].

Long ncRNAs (lncRNAs), as their name suggests, are not translated into proteins and are defined as being longer than 200 base pairs (bp) [8]. The *SNHG14* lncRNA is particularly long at 3700 kilobases (kb) and is classified as a macro ncRNA. LncRNAs can be found spliced in the cytoplasm or primarily unspliced in the nucleus, which contributes to their typically shorter half-life compared to messenger RNA (mRNA) [8, 55].

**Figure 2.**
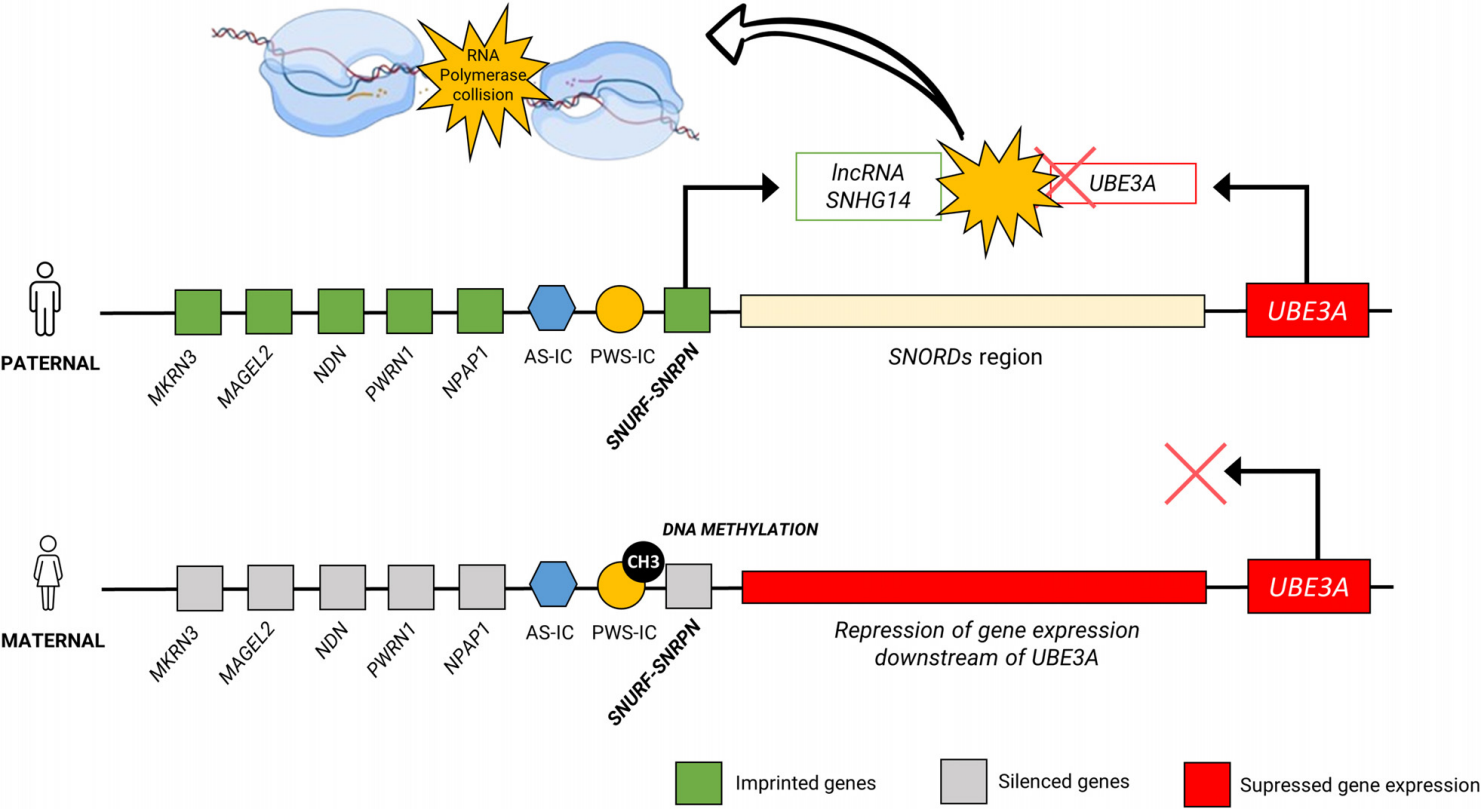
**Schematic of the epigenetic imprinting regulation in Angelman syndrome, located in chromosome 15q11-q13 of neuron cells and the plausible theory of silencing mechanism of paternal *UBE3A* gene.** UBE3A: Ubiquitin-protein ligase E3A.

In mouse neurons, the *Snhg14* lncRNA is expressed only from the paternal allele, while *Ube3a* is expressed only from the maternal allele. The proposed model for *Ube3a* silencing on the paternal chromosome in neurons is known as the collision model. This model suggests that during *Snhg14* transcription, the transcriptional machinery extends into the *Ube3a* termination region. This overlap leads to transcriptional collisions between RNA polymerases, causing truncated elongation and subsequent degradation of the paternal *Ube3a* transcript [8] (Figure 2). Therefore, inhibiting *Snhg14* could be a potential therapeutic strategy to unsilence the paternal copy of *UBE3A* [56]. An American research group observed that mice with a maternally deleted Ube3a allele (m−/p+) treated with topotecan, a Topoisomerase I inhibitor, exhibited increased *UBE3A* expression compared to wild-type mice [57]. This finding suggests that Topoisomerase I inhibition disrupts transcriptional progression along the *Snhg14* region. Since *Snhg14* is not expressed from the paternal chromosome, this disruption allows UBE3A to be expressed. However, topotecan and other Topoisomerase I inhibitors affect more than just the *UBE3A* locus on chromosome 15q11-q13, limiting their specificity and making them a less attractive therapeutic option for humans.

As evidenced by the information reported above, literature on epigenetic mechanisms in AS remains limited. However, elegant studies on NDDs with clinical similarities to AS have provided valuable insights (reviewed in [42]). Research on children with neurodevelopmental defects indicates that DNA methylation and histone modification are crucial for normal brain development [58]. Moreover, proper transcriptional regulation through chromatin remodeling, as well as the action of ncRNAs, such as miRNAs and lncRNAs, plays a crucial role in neurodevelopmental processes [53, 59–62].

**Figure 3.**
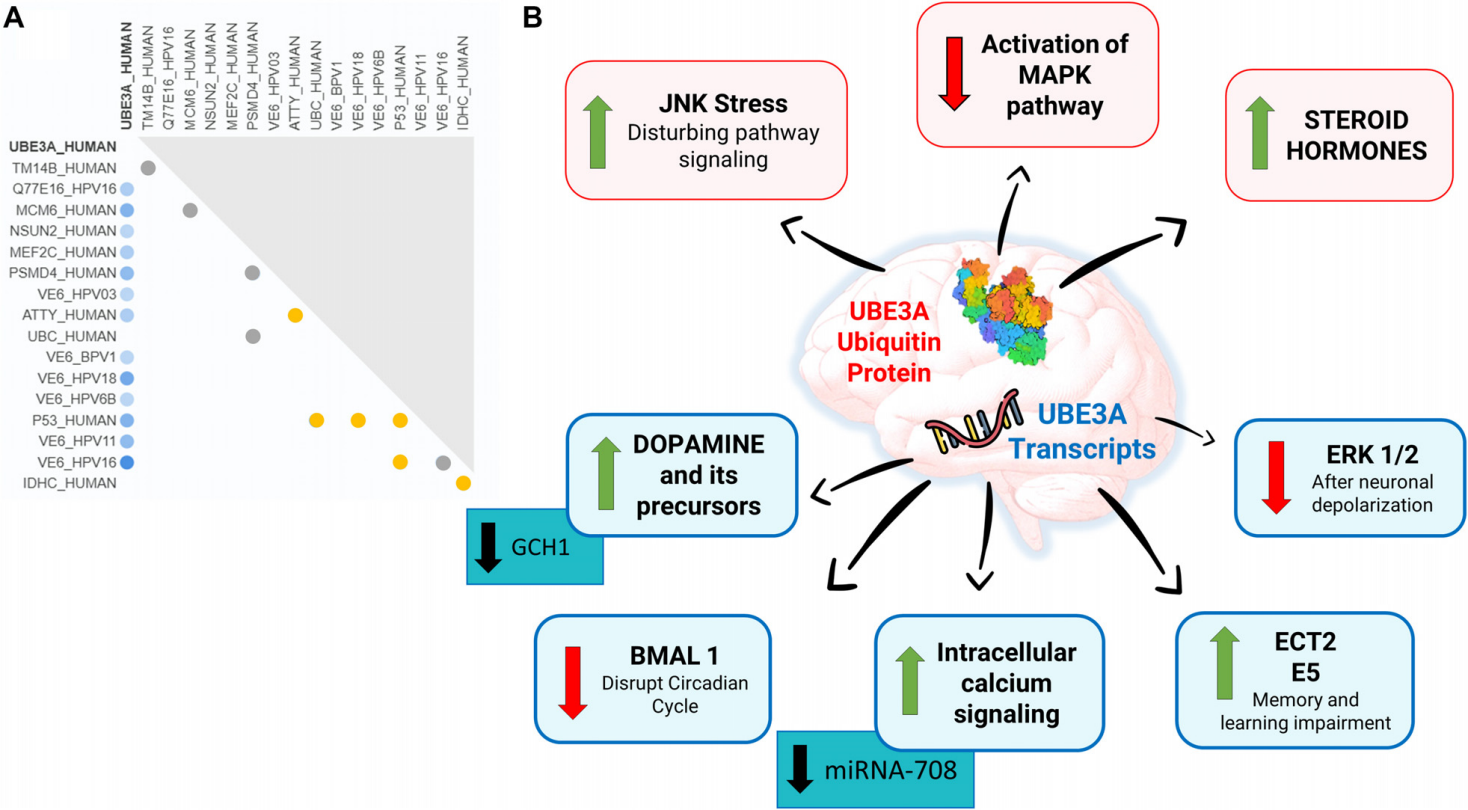
(A) UBE3A protein interactions based on UniProt data. Blue circles indicate interactions associated with Angelman syndrome, yellow circles indicate associations with other diseases, and gray circles indicate interactions with no known disease association. (Modified from: https://www.uniprot.org/uniprotkb/Q05086/entry#interaction.) (B) The absence or deficiency of UBE3A ubiquitin protein and transcripts in the nervous system disrupts several cellular functions and negatively affects neuronal cell physiology. UBE3A: Ubiquitin-protein ligase E3A.

Currently, the most advanced approach for treating AS involves the use of antisense oligonucleotides (ASOs) targeting a conserved region of *SNHG14*. This strategy represses SNHG14 transcription, thereby enabling the expression of paternal *UBE3A* [63, 64]. Dindot and colleagues achieved promising results with this ASO in both *in vitro* and *in vivo* studies using monkey specimens [63]. This therapeutic approach is now being evaluated in clinical trials (GeneTx NCT04259281; Roche NCT04428281). However, beyond the challenge of determining the optimal timing for restoring functional paternal *UBE3A* expression in human trials, another critical consideration is *UBE3A’s* interaction with other proteins and pathways that may be disrupted by its absence in the brain (Figure 3A) [64]. These interactions must be carefully assessed when developing new therapies.

### The homeostatic level of *UBE3A* expression is critical to maintaining normal neuronal function

The ubiquitin-proteasome system (UPS) is a major pathway for intracellular protein degradation in eukaryotic cells, involving a large group of post-translational modification proteins [3, 65]. Ubiquitination plays a crucial role in maintaining cellular homeostasis by regulating various functions, including proteasomal degradation, selective autophagy, cell signaling, endocytosis, receptor trafficking, DNA damage response, cell cycle control, and programmed cell death [3]. The *UBE3A* gene encodes a ubiquitin-protein E3 ligase, a ∼100 kDa enzyme involved in the three-step ubiquitination process, which requires a cascade of three enzymes: E1, E2, and E3 [3, 15]. First, E1 enzymes activate ubiquitin (Ub) by attaching it to E2. Then, E3 ligases recognize the E2-Ub complex and facilitate the transfer of Ub to the target protein [66].

The E3 ubiquitin ligase is responsible for ensuring the specificity of the ubiquitination process, so it is plausible to have a large number of these enzymes—more than 800 have been identified so far—while only a small portion of E1 activating enzymes and E2 conjugating enzymes exist [65, 66]. E3 ligases can be classified into four types: the most common Really Interesting New Gene (RING) finger type and the Homologous to the E6-associated protein (E6-AP) Carboxyl Terminus (*HECT*) type, as well as the less common U-box and RBR types [66].

The UBE3A protein was originally known as E6-AP because it interacts with the Human Papillomavirus (HPV) E6 oncoprotein to degrade the cell cycle protein p53 [67]. However, later research revealed that this degradation occurs only in the presence of and in association with the E6 viral oncoprotein [68].

In 1998, Jiang and colleagues established a mouse model for AS [69] by completely knocking out the maternal *UBE3A* gene at exon 2. These mice exhibited key clinical features of AS, including motor disabilities, seizures, sleep disturbances, and learning and memory deficits. Additionally, they showed increased cytoplasmic p53 levels in postmitotic Purkinje cells in m−/p+ mice. Considering Cooper’s findings in 2003 [68], it is possible that E6-AP plays a significant role in regulating p53 levels *in vivo* by utilizing a substitute molecule for E6, as previously suggested by Jiang et al. [69].

The UBE3A protein plays a crucial role in target protein recognition, ensuring specificity in the ubiquitination process. Therefore, its absence or deficiency in the nervous system can be highly detrimental to neurons. While UBE3A deficiency leads to AS, elevated levels of the protein are associated with autism spectrum disorder (ASD) [41]. The duplication of the 15q11-q13 chromosome region increases UBE3A levels, exacerbating ASD symptoms—a phenomenon observed in rodent models [69]. This highlights that the precise regulation of UBE3A is critical in determining the clinical outcomes of affected individuals.

The gene *UBE3A* plays a crucial role in gene expression by generating several transcription factors that interact with various molecules. In a 2011 study using Drosophila flies, Ferdousy and colleagues demonstrated that *UBE3A* (Dube3A) acts as a transcriptional coactivator, upregulating GTP cyclohydrolase I (GCH1). Consequently, the absence of Dube3A in Drosophila leads to increased levels of dopamine and its precursors [70].

Additionally, evidence suggests that *UBE3A* transcription is essential for maintaining the circadian clock by regulating the transcription factor Brain and Muscle ARNT-Like 1 (*BMAL1*). Gossan and colleagues have demonstrated that *UBE3A* levels *in vivo* are critical for regulating the circadian system in both mammals and flies. Their findings indicate that, in the absence of UBE3A, BMAL1 protein levels are higher in wild-type rodents [71]. Moreover, *UBE3A* interacts with the factors ECT2 (Epithelial Cell Transforming Factor) and Ephexin V (E5). These molecules regulate Rho GTPases, which are essential for maintaining proper dendritic spine density and, consequently, neuronal plasticity in the brain. The loss of UBE3A expression disrupts the regulation of these molecules, potentially leading to memory and learning impairments [72].

The UBE3A protein also functions as a coactivator for steroid hormone receptors, including progesterone, estrogen, androgen, glucocorticoid, retinoic acid receptor-α, and thyroid hormone receptors [73]. A deficiency or improper regulation of functional UBE3A in the brain can lead to the accumulation of its target proteins, potentially contributing to the pathogenesis of AS (Figure 3B).

### The impact of UBE3A deficiency on cellular pathways

Studies demonstrate that UBE3A levels influence key cellular pathways, including cAMP, MAPK, c-Jun N-terminal kinase (JNK), and extracellular signal-regulated kinase (ERK). Filonova and colleagues (2015) showed that in an AS mouse model (Ube3a m−/p+), the activation of p44/p42 ERK1/2 is impaired following neuronal depolarization. This finding indicates that the absence of UBE3A reduces MAPK activation in the brain [74], which in turn affects synaptic plasticity and memory formation. Additionally, the lack of UBE3A leads to increased JNK activity—a stress signaling pathway—and a decreased p-ERK/ERK ratio in heterozygous (m−/p+) mice compared to wild-type [75]. JNK activation in the brain may contribute to neurodegeneration by phosphorylating c-Jun, thereby triggering neuronal death. This suggests that JNK signaling inhibitors could be a promising treatment target (Figure 3B). Since UBE3A is a key ubiquitin-protein ligase responsible for degrading intracellular proteins, its absence may result in the accumulation of various substrates, directly affecting cell signaling.

Vatsa and colleagues also demonstrated in a mouse model of AS that in rodents with *Ube3a* (m−/p+), miRNA-708 is downregulated in the brain. Since miRNA-708 plays a crucial role in regulating intracellular calcium homeostasis—essential for neuronal function—its deregulation leads to an abnormal increase in calcium signaling in AS mice. This disruption may, in turn, affect synaptic plasticity in the context of AS [76].

In the AS mouse model (m−/p+), there is a disruption in neuroplasticity, specifically in long-term potentiation (LTP) within the hippocampus [69, 77]. Maintaining basal synaptic plasticity and transmission involves a coordinated process between adenosine G protein-coupled receptors (GPCRs), particularly the adenosine A2A receptor (A2AR) and A1 receptor (A1R) [78–80]. Under normal conditions, A2AR expression in the brain is low compared to A1R. However, during high-frequency synaptic activity, A2AR is upregulated, meaning it is recruited only during intense nerve stimulation that induces synaptic changes, such as LTP [79]. Given this, evidence suggests that A2AR may play a role in the pathophysiology of AS. In 2020, a Portuguese research group investigated whether blocking A2AR could improve memory dysfunction and synaptic plasticity. They found that AS mice (*Ube3a* m−/p+) exhibited impaired hippocampal-dependent learning and memory in the Morris Water Maze, along with increased A2AR expression in hippocampal tissue. Chronic treatment with a selective A2AR antagonist restored hippocampal-dependent learning strategies and rescued LTD deficits [81].

If the absence of *Ube3a* in rodents leads to an accumulation of A2AR, it is plausible that the lack of UBE3A in humans could also interfere with the expression of adenosine receptors in the brain. In fact, A2BR plays a crucial role in energy regulation in the brain, participating in cAMP signaling in astrocytes to modulate their metabolic activation via the cAMP–PKA signaling pathway. Moreover, an upregulation of this adenosine receptor in the brain has been observed to support this function [82]. Therefore, investigating the role of adenosine receptors in UBE3A models could provide valuable insights into the pathophysiology of AS and potentially lead to new combination treatment approaches.

### Future perspectives

Genetic imprinting is one of the most fascinating aspects of molecular genetics, and AS is a notable imprinting disorder affecting up to 500,000 people worldwide. Over the past 60 years, significant progress has been made in understanding its molecular and genetic mechanisms. However, an effective treatment remains elusive.

Currently, the most studied therapeutic approach focuses on inhibiting *SNHG14*, either directly through ASOs or indirectly via topotecan-mediated inhibition. Despite this progress, concerns remain regarding the specificity and efficacy of this strategy in *in vivo* models, as well as the optimal timing for restoring functional paternal *UBE3A* expression in human clinical trials.

## Conclusion

In conclusion, elucidating the molecular mechanisms behind the silencing of the paternal *UBE3A* allele is crucial for addressing the root cause of AS and restoring functional UBE3A protein expression in affected individuals. However, given UBE3A’s extensive interactions with other proteins in the brain, it is equally important to consider modulating abnormal signaling pathways for a more effective combination therapy. Additionally, investigating receptor expression imbalances in neuronal cells of AS models may be key to unlocking promising new treatment targets.
